# Refractory Paroxysmal Sympathetic Hyperactivity Following Traumatic Intracerebral Hemorrhage

**DOI:** 10.7759/cureus.19086

**Published:** 2021-10-27

**Authors:** Matea Malinovic, Kristine Kallenberger, Justin Sandall

**Affiliations:** 1 Anesthesiology, University of Kansas School of Medicine Wichita, Wichita, USA; 2 Anesthesiology, Memorial Surgery Center, Tulsa, USA

**Keywords:** intracerebral hemorrhage, traumatic brain injury, paroxysmal sympathetic hyperactivity, dysautonomia, brain injury, autonomic nervous system diseases

## Abstract

Paroxysmal sympathetic hyperactivity (PSH) is a complex and underrecognized phenomenon involving increased sympathetic activity leading to tachycardia, tachypnea, and hypertension. The frequency of nonrecognition is due to a lack of diagnostic criteria. In addition, the lack of evidence-based treatments has further complicated approaches to management. This case describes a patient who presented with a new-onset seizure and intracerebral hemorrhage requiring burst suppression and decompressive craniotomy to attenuate intracranial hypertension (ICH). The patient continued to display paroxysmal dysautonomia requiring a multimodal regimen for control of episodes. He demonstrated neurological improvement and complete resolution of dysautonomic activity prior to being discharged to a rehabilitation facility. A delayed diagnosis, ICH, and refractory PSH status postdecompressive craniotomy presented unique challenges. Given that the number of episodes of paroxysmal “storms” directly correlates with morbidity and mortality, early detection is critical, and lack of recognition makes this a difficult disorder to diagnose and manage.

## Introduction

Intracerebral hemorrhage is defined as blood in the brain parenchyma with potential extension into the ventricles [1]. Traumatic brain injury (TBI) is one of the most prevalent causes of intracerebral hemorrhage but nontraumatic causes such as hypertension, amyloid angiopathy, arteriovenous malformation, and intracranial aneurysm exist [1]. Paroxysmal sympathetic hyperactivity (PSH) is a condition associated with acquired brain injuries (ABI) that can lead to dire consequences if not recognized. PSH can exacerbate morbidity and mortality, is often difficult to treat, and has been described for decades but has been underrecognized because of its numerous reference terms [2]. In 2014, a list of clinical and diagnostic criteria was developed to define PSH [3]. However, the diagnosis and treatment of PSH still remain challenging due to differences in patient presentations and responses to treatment. Proposed treatments have yet to be studied in large-scale clinical trials and pose additional difficulties in determining adequate treatment for dysautonomic episodes due to the variability in patient response [4]. Our case presents a patient who had a late diagnosis of PSH due to initially unrecognized TBI, further complicated by refractory dysautonomic episodes after decompressive craniotomy, which were abated using a multimodal regimen. Written informed consent was obtained from the patient. This article was previously presented as a presentation at the Neuroscience Case Conference on February 3, 2017.

## Case presentation

**Figure 1:**
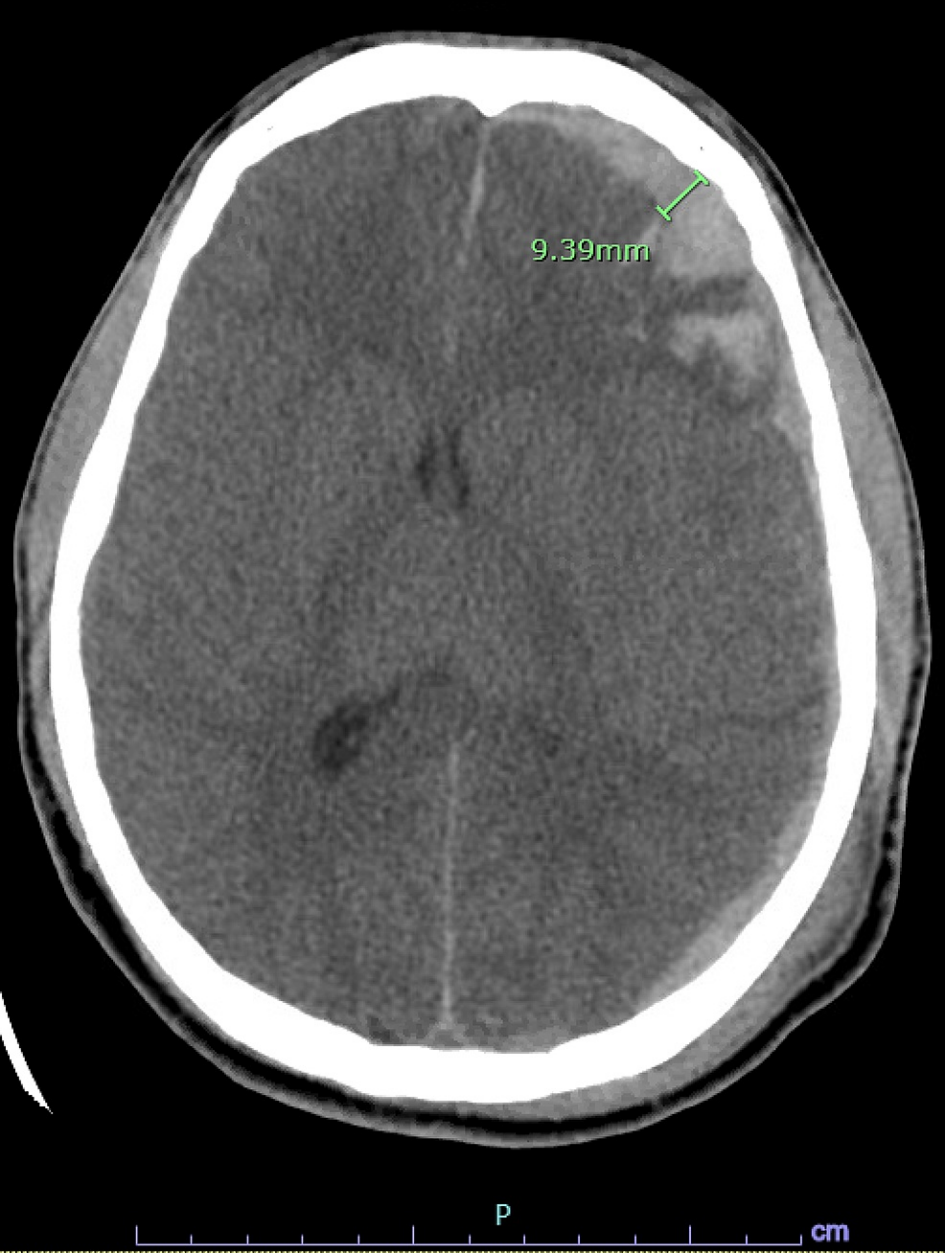
CTA head/neck. Intraparenchymal hemorrhage and 9 mm subdural hemorrhage were visualized in the left frontal lobe and left temporal lobe. CTA: computed tomography angiography.

**Figure 2:**
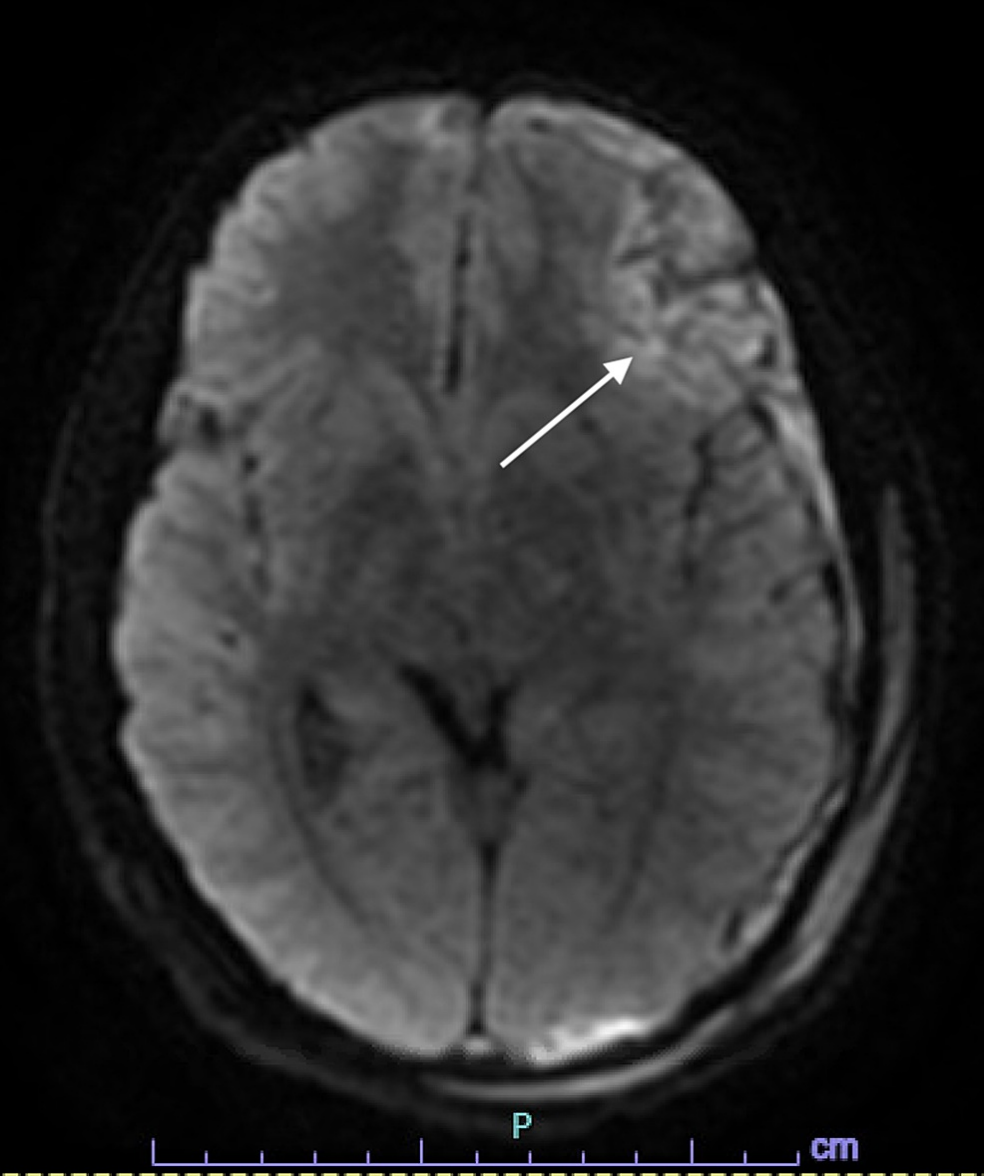
MRI brain diffusion-weighted image. A 4.0 cm × 2.9 cm left frontal intraparenchymal hemorrhage (indicated by the arrow) with subarachnoid hemorrhage and subdural hematoma components was evident.

**Figure 3:**
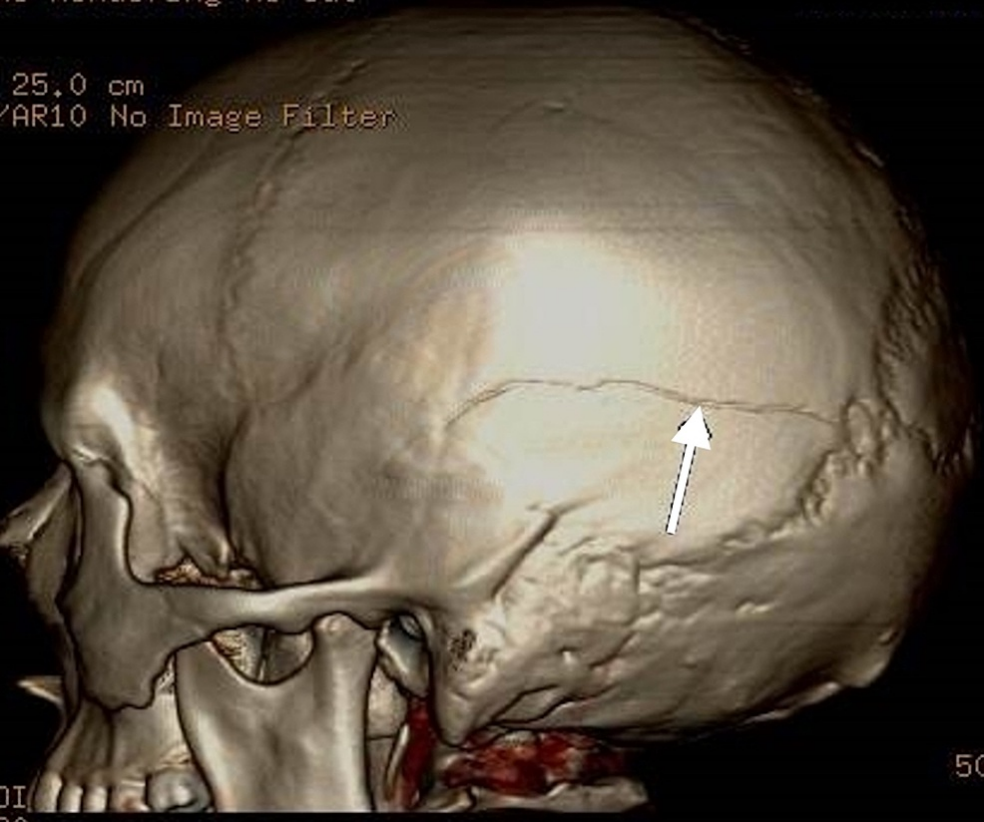
CTA head/neck. A left nondisplaced fracture (indicated by the arrow) in the confluence of the parietal and temporal bones was visualized. CTA: computed tomography angiography.

**Figure 4:**
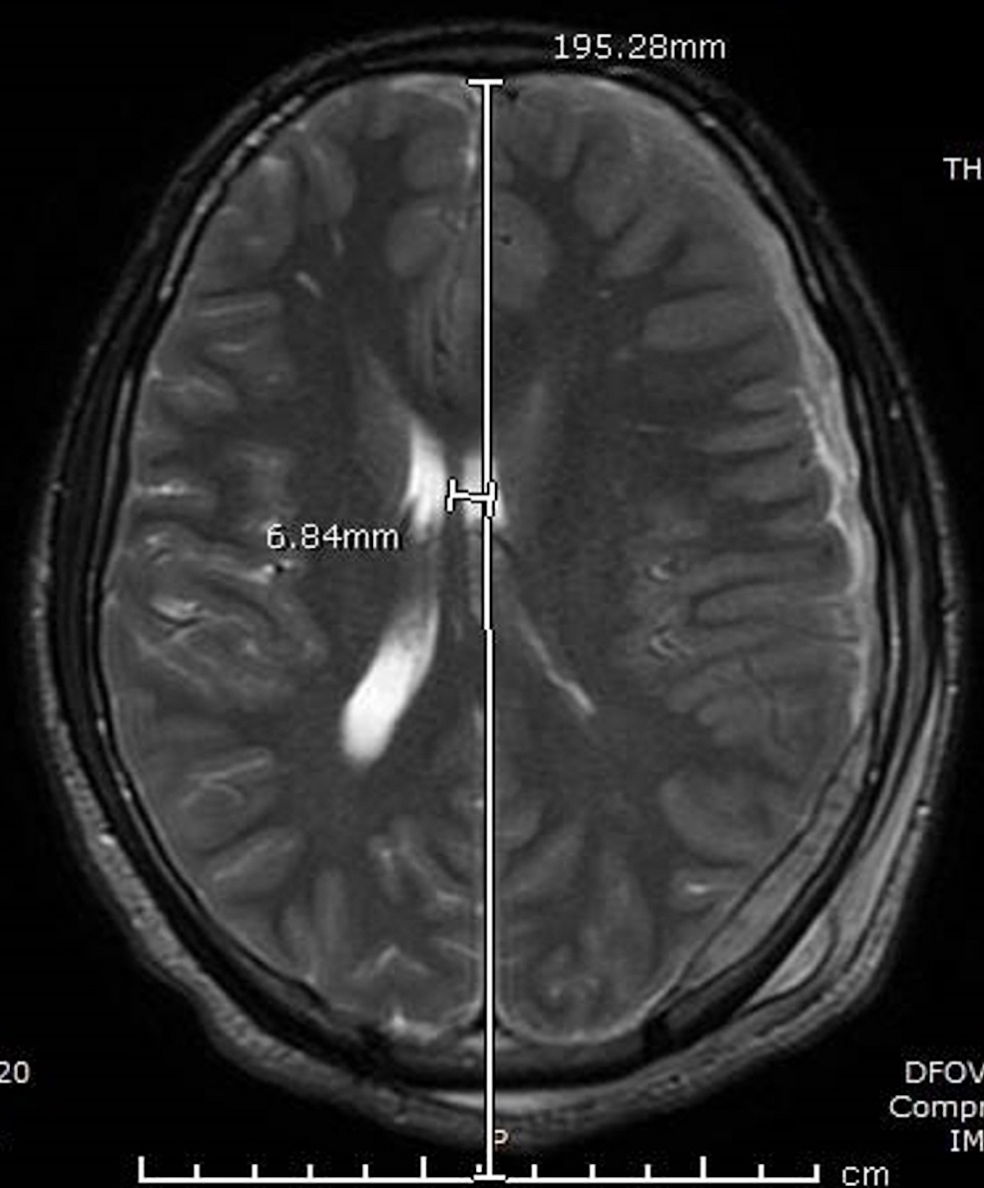
MRI brain axial T2-weighted. The presence of cerebral edema with a 6 mm left-to-right midline shift was evident.

An 18-year-old male presented to an outlying emergency department after having a seizure at his workplace. Prior to transfer, he was intubated due to increasing agitation, and a CT head without contrast was performed at the outside hospital. On arrival to our facility, he initially underwent computed tomography angiography (CTA) head/neck followed by MRI brain on the day of admission. Initial vital signs included a respiratory rate of 15, blood pressure 133/66, oxygen saturation of 100%, and a heart rate of 84. He was moving all extremities, but he would not wake or follow commands. His Glasgow Coma Score (GCS) was 7T. A nicardipine drip was initiated for control of hypertension, and he was placed on levetiracetam for seizure prophylaxis. Close contacts of the patient denied a history of head trauma, and the urine drug screen was negative for stimulants. CTA head/neck indicated intraparenchymal (IPH) and 9 mm subdural hemorrhage (SDH) in the left frontal lobe and left temporal lobe (Figure 1). MRI brain diffusion-weighted imaging revealed a 4.0 cm × 2.9 cm left frontal IPH with subarachnoid hemorrhage (SAH) with SDH components (Figure 2). A left nondisplaced fracture in the confluence of the parietal and temporal bones was visualized, indicative that blunt trauma likely accounted for the intracranial bleeds (Figure 3). Additionally, the presence of cerebral edema with a 6 mm left-to-right midline shift was appreciated (Figure 4).

 

Measures to reduce intracranial pressure (ICP) were initiated, including elevating the head of the bed to greater than 30 degrees, hyperosmolar therapy, and propofol sedation to a Richmond Agitation-Sedation Scale goal of −1 to −2 with daily awakening trials. On hospital day 2, neurosurgery placed an external ventricular drain to monitor ICP and allow for cerebrospinal fluid diversion. The presence of intracranial hypertension (ICH) was confirmed with an ICP of 22 mmHg. On hospital day 3, the patient began exhibiting cerebral dysautonomia with intermittent hyperthermia (Tmax 39.0°C), tachycardia (HR 110s-140s), tachypnea (20s-30s), and hypertension (systolic blood pressure >150 mmHg despite being on nicardipine infusion). ICH persisted despite maximizing hyperosmolar therapy and deepening sedation. Due to the failure of standard medical treatments, burst suppression was initiated and maintained using a pentobarbital infusion while maintaining cerebral perfusion pressure with hemodynamic manipulations as needed. After a week of induced coma, the pentobarbital infusion was decreased while simultaneously adding dexmedetomidine and fentanyl drips to attenuate sympathetic storming. While the neurosurgery team was consulted on admission, craniotomy was not performed initially since the patient was maintaining adequate cerebral perfusion pressures. The patient’s ICP was labile but stable during burst suppression with pentobarbital. However, during attempted pentobarbital weaning, he experienced persistent elevations in ICP. Decompressive craniotomy was then performed on hospital day 11 to stabilize ICP as well as allow for the reduction in sedative medications. Pentobarbital was discontinued postoperatively.

After craniotomy, the patient continued to have refractory cerebral dysautonomia as evidenced by tachycardia, hyperthermia, diaphoresis, tachypnea, and decorticate posturing. The storms continued to be attenuated with scheduled propranolol and fentanyl and dexmedetomidine drips. Despite aggressive management of hyperthermia, his temperature increased to 40°C. Intravascular cooling was initiated for seven days with a goal of normothermia (37°C). This led to shivering; therefore, meperidine therapy was initiated. Clonidine and enteral morphine were added, while fentanyl and dexmedetomidine drips were titrated off. A multimodal regimen including clonidine, morphine, bromocriptine, propranolol, and gabapentin proved successful, and the patient did not have storm activity prior to discharge. Prior to discharge, he demonstrated normal motor and sensory function and was able to speak clearly with a Passy Muir speaking valve (Passy Muir Inc., Irvine, CA, USA). After approximately one month in intensive care, the patient was transferred to an inpatient rehabilitation center.

## Discussion

PSH results from neuronal damage that activates the autonomic nervous system and is characterized by an increase in catecholamine release [5]. A multitude of clinical features including tachypnea, tachycardia, diaphoresis, hypertension, hyperthermia, ventilator desynchrony, and posturing, along with the myriad of terms used to describe PSH, have led to misdiagnosis and lack of recognition of PSH [2,5]. In addition, lack of recognition has impeded research on diagnostic approaches and treatment [5]. However, earlier recognition of the condition has decreased morbidity [2]. While it has been documented in ABI such as ischemic stroke and intracerebral hemorrhage [2], TBI accounts for 79.4% of PSH cases [6]. It is considered a diagnosis of exclusion due to the overlapping characteristics associated with conditions such as seizures and sepsis [2]. Conditions such as dehydration, further seizures, pulmonary embolism, and drug-induced diseases were excluded in this patient. The patient did have a positive mini bronchoalveolar lavage for methicillin-susceptible *Staphylococcus aureus,* which was successfully treated with nafcillin. Blood cultures remained negative through the hospital stay. Baguley et al. proposed a clinical and diagnostic likelihood scale to be used as an assessment for evaluating the probability of a PSH diagnosis [3]. Our patient’s medical course was complicated by an initially unrecognized blunt force injury leading to the workup of spontaneous intracerebral hemorrhage instead of an immediate diagnosis of TBI. This led to a delay of recognition of PSH episodes, potentially resulting in their refractory nature. Retrospectively, this patient’s PSH assessment score was calculated as 23 points indicating that PSH diagnosis likelihood was probable.

In addition to the lack of recognition for the diagnosis of PSH, there is a lack of guidelines for the classification of severity of storming events and evidence-based treatment algorithms [2]. However, several treatment options have been explored for PSH with the aim to inhibit the sympathetic outflow and the afferent sensory process [7]. The mode of action varies, including centrally acting agents to decrease sympathetic discharge to peripherally acting agents to decrease the effect of catecholamines from the sympathetic nervous system. Reduction of external stimuli and pharmacological therapy designed to either prevent or terminate the dysautonomic episode is critical in treatment [8]. The most common treatment options utilized include baclofen, benzodiazepines, gabapentin, bromocriptine, opioids, α2 agonists, and nonselective β antagonists [5]. Morphine and benzodiazepines are utilized initially because of their rapid effects resulting in the resolution of episodes [5,8]. Our patient went through several therapies with minimum effects. His initial therapy with propofol was ineffective. His course was further complicated by elevations in ICP leading to burst suppression with pentobarbital infusion and decompressive craniotomy. However, his PSH episodes were refractory and continued postoperatively. Scheduled propranolol was utilized. Propranolol was the β antagonist selected due to its nonselective lipophilic nature and ability to cross the blood-brain barrier. Propranolol has also been shown to decrease sympathetic effects. It has been shown as the preferred β antagonist for PSH with effects of improving mortality and was previously studied utilizing the oral route [5-7]. Ultimately, propranolol, clonidine, bromocriptine, and gabapentin provided an element of preventive therapy for PSH episodes with the greatest effect on tachycardia and hypertension for our patient [8].

While analgesics such as acetaminophen have been used in the small observational cohort study by Pozzi et al., they have not been shown to have statistically significant efficacy in controlling PSH episodes [9]. Our case presents a patient in which acetaminophen and cooling blankets were ineffective in controlling temperatures and an intravascular cooling device was placed with the successful outcome of controlling body temperature. Intravascular cooling resulted in shivering and meperidine was administered as needed. The meperidine effects via the α2B adrenoceptor work synergistically with dexmedetomidine and have been incorporated into the Columbia anti-shivering protocol [10]. This multimodal combination eventually proved successful with neurological improvement and resolution of PSH episodes leading to recovery and discharge to inpatient rehabilitation.

## Conclusions

Our case presents a difficult diagnostic and therapeutic dilemma. Unrecognized TBI led to a delay in diagnosis, and the patient ultimately developed refractory ICH and cerebral dysautonomia despite maximal medical and surgical therapy. Although surgical decompression resulted in the resolution of persistent ICH, dysautonomia continued and required multiple centrally acting agents to ameliorate and treat the underlying etiology. The diagnosis of cerebral dysautonomia is often delayed due to the presence of confounding factors or etiologies that can share similar characteristics. Moreover, there is a lack of data to help guide appropriate therapy once it is diagnosed, and further research and recommendations need to be conducted regarding etiology, prevention, and treatment modalities of PSH. Early recognition is key to reducing morbidity. We present a patient with a late diagnosis of PSH and refractory episodes who required a multimodal regimen for treatment which ultimately proved effective.
